# A taxonomic schema of potential pitfalls in clinical variant analysis based on real-world evidence

**DOI:** 10.1371/journal.pone.0295010

**Published:** 2023-11-30

**Authors:** Adam Coovadia, Luigi Boccuto, Yenui Chang, Jane DeLuca

**Affiliations:** College of Behavioral, Social and Health Sciences, Clemson University, Clemson, South Carolina, United States of America; Umass Chan Medical School, UNITED STATES

## Abstract

The classification and interpretation of genetic variants associated with genetic diseases have been shown to vary between clinical genetic laboratories. This can lead to errors introduced in the interpretation and public presentation of genetic findings in the literature and available databases. This qualitative study utilizes real-world evidence to introduce a taxonomic schema of potential pitfalls associated with public and commercial resources commonly used for sequence variant analysis. Databases, articles and other resources continue to evolve over time. A modified and expanded version of Reason’s Model of Human Error with respect to variant analysis is proposed and discussed. This study complements professional standards and published recommendations of interpretive considerations associated with variant analysis and expands the scope of professional competency.

## Introduction

Variant analysis is the process of classifying variants in an effort to determine their clinical significance. The American College of Medical Genetics and Genomics (ACMG) and the Association for Molecular Pathology (AMP) [1] have been providing published guidelines and recommendations for the interpretation of genetic variants. While discretionary deviations are permitted, a germline variant is typically classified according to a continuous spectrum with distinct delineations of being benign, likely benign, uncertain/unknown significance, likely pathogenic, or pathogenic. However, there is no recommended singular approach to the analytical process. Additionally, the College of American Pathologists (CAP) (the United States’ accrediting regulatory body for Clinical Laboratory Improvement Amendments (CLIA) certification of genetic laboratories) does not require laboratories to have a standard operating procedure for the process of performing variant analysis [2]. Consequently, each clinical laboratory wields full discretion as to the specifics of how and when to perform variant analyses for a given germline variant. Once classified, interpreted and reported to the patient or client, the sharing or public reporting of the variant’s classification is voluntary. Of the reporting institutions, it is known that clinically significant differences exist between the classifications of germline variants. ClinGen, a National Institutes of Health (NIH) funded resource dedicated to defining the clinical relevance of genes and variants for use in precision medicine and research, reports that thousands of variants have discrepant assertions [3]. Arguably, the most disconcerting type of variant classification discordance contains many thousands of conflicting assertions that differ between benign and pathogenic designations by different institutions. However, a study of likely pathogenic/pathogenic variants in ClinVar without discordance between reporting laboratories showed that 40% of the likely pathogenic/pathogenic variants reviewed should be downgraded to benign, likely benign, or variants of uncertain significance according to ACMG standards [4]. While there are studies and reports of the potential pitfalls encountered in the process of gene curation and variant interpretation, this novel qualitative study utilizes real-world data derived from the routine clinical genetic analysis of germline variants according to the ACMG/AMP recommendations and guidelines.

Real-world data can be defined as data collected in a non-randomized controlled trial setting [5]. It refers to observational data that can be derived from sources such as electronic health records, patient registries, and billing activities. Real-world evidence is a body of facts derived from real-world data. This type of evidence may yield more information and serve as a better representation of a studied population than that of a controlled study [6, 7]. In this study, real-world data from a sample of germline variants were collected to build evidence of actual variant analysis pitfalls.

A taxonomy can be defined as information architecture or a knowledge organization system [8, 9]. Derived from the Greek word *taxis* meaning arrangement, order, or noun of action “to arrange”, and borrowed from the French, *taxonomie*, a taxonomy is a systematic classification, especially in relation to general laws and principles of a particular science or subject [10]. Taxonomic elements that enable a taxonomic process include setting-controlled vocabularies (designated terms or phrases), developing facets (grouping of content from different perspectives), assigning categories (aka classes), and establishing ontologies (relationships and hierarchy).

The purpose of this study was to examine real-world data of variants using existing evidence to propose a taxonomic schema of potential pitfalls found in public and commercial sources used in the analysis of potentially clinically relevant variants.

## Materials and methods

Real-world variant data were provided for this study by a Clinical Laboratory Improvement Amendments (CLIA) certified, College of American Pathologists (CAP) accredited molecular genetics laboratory. The germline variants were detected from DNA sequence-based tests for inherited diseases such as cystic fibrosis, cholestasis, short stature, inherited cancers, autism, epilepsy, and other conditions. Variants detected were subject to the laboratories’ proprietary bioinformatics standard operating procedure to ensure accurate calls and filtered according to the following specifications: a) Non-reference call is 35–70% (i.e., heterozygous) or non-reference call is 95–100% (i.e., homozygous); b) 0% call for deletion; c) 0% call for duplication; d) coverage is 15X or more; e) No pseudoexon detected. The process of variant analysis was blinded to patient information including the phenotype and family history. The variants were interrogated using ACMG/AMP recommended resources and tools (Table 1).

**Table 1:** Ancillary data sources.

Ancillary Data Source
dbSNP v153, 154 (https://www.ncbi.nlm.nih.gov/snp/)
HGMD Pro® v2019.2, 2019.3, 2019.4, 2020.1, 2020.2 (https://www.ncbi.nlm.nih.gov/snp/)
gnomAD (https://gnomad.broadinstitute.org/)
Google Scholar (https://scholar.google.com/)
Google (https://www.google.com/)
PubMed (https://pubmed.ncbi.nlm.nih.gov/)
Mastermind® (https://mastermind.genomenon.com/)
Leiden Open Variation Database (https://www.ncbi.nlm.nih.gov/clinvar/)
ClinVar (https://www.ncbi.nlm.nih.gov/clinvar/)
ClinGen (https://clinicalgenome.org/)
UniProt (https://www.uniprot.org/)
National Center for Biotechnology Information Gene (https://www.ncbi.nlm.nih.gov/gene/)
Online Mendelian Inheritance in Man (https://www.ncbi.nlm.nih.gov/omim)
HGNC (https://www.genenames.org/)
Mutalyzer v2.0.32 (https://mutalyzer.nl/)
Combined Annotation Dependent Depletion v.1.6 (https://cadd.gs.washington.edu/snv)
1000 Genomes Phase 3/Ensemble Genome Browser Phase 3 (https://uswest.ensembl.org/index.html)
Cystic Fibrosis Mutations Database (http://www.genet.sickkids.on.ca/)
CFTR-France (https://cftr.iurc.montp.inserm.fr/cftr/)
Clinical Functional Translation of CFTR (https://cftr2.org/)
Deafness Variation Database (http://deafnessvariationdatabase.org/)
Mutation Database Autosomal Recessive Polycystic Kidney Disease (http://www.humgen.rwth-aachen.de/)
Rare Exome Variant Ensemble Learner (Pre February 5th, Post February 5th Updates) (https://sites.google.com/site/revelgenomics/about)

Real-world data and evidence were derived from the identification of discrepancies encountered during the routine analysis of the germline variants associated with the aforementioned clinical genetic tests using public and commercial genomic analytical tools (Table 1). A discrepancy was defined as inconsistent, discordant, or conflicting variant information that could influence the clinical interpretation and or inhibit the ability to reconcile information on the variant and/or its gene.

Examples of discrepancies were collected as images using presentation software along with descriptive information including the specific variant queried. For the purpose of copyright considerations, only the description of the discrepancies is provided (see S1 Dataset). Data were collected over a period of 12 months (from August 2019 to August 2020) until saturation of the type of discrepancy was achieved and/or the study data collection deadline was reached. The data were subsequently analyzed using inductive conventional content analysis–a process used to determine the presence of themes or concepts within qualitative data [11–14]. The discrepancies were described, annotated in a tabular format, and subjected to merit review and multiple iterations of classification and or categorization. Semantic categorization was coupled with a colloquial phasing to enhance communication, meaning, and future practice and inquiry. A review of the discrepancies and the categories was performed by a clinical genetics researcher not associated with the laboratory. The defined categories served as the controlled terms necessary for building a taxonomic model consistent with the defined categories. A taxonomic schema was built using an iterative process assessing the relationship of the evidence categories. This research does not qualify for submission to an IRB or REC in any of the researchers’ jurisdictions.

## Results

Examples of discrepancies were collected, analyzed, and described (see S1 Dataset). Sources of data discrepancies were provided and included hyperlinks as evidence. Additional supporting information was provided as evidence, when appropriate. The examples were organized according to eleven primary categories of discrepancies (see Table 1). A facet taxonomic structure was determined.

A discrepancy can be defined as a lack of compatibility or dissimilarity between two or more facts. While the word pitfall is often used in a broad sense of things that could go wrong in a process, a pitfall can be defined as a hidden or unsuspected danger or difficulty [15]. The discrepancies identified in this study serve as evidence of potential pitfalls in the variant analysis. A discussion of the eleven distinct categories is provided for each of the identified potential pitfalls in this study. The evidence of each category is available in the (see S1 Dataset).

### The Presumed Typo

In reference to mistakes in manual typesetting, a Presumed Typo is defined as a deduced misprint or typographical error that is assumed to be unintentional. This category entailed examples of inappropriate nomenclature, incorrect coordinates, inaccurate protein predictions and descriptions, numerical transpositions, incorrect assertions of zygosity, and errors in the reporting of functional information. The real-world evidence indicated that the Presumed Typo can appear in the text, tables, and supplements of recent and decades-old peer-reviewed published articles and within reputable public and commercial databases. The Presumed Typo may impact the total tally of patient accounts thereby influencing the prevalence of the variant within an affected population. A single Presumed Typo may involve many variants. A Presumed Typo can persist and be undetected and or unacknowledged. It can be inferred that a Presumed Typo can lead to a failure of the variant and or related information being found within an article, thereby misrepresentation, devaluation or dismissal of the article’s findings could be perpetuated because of the erroneous information.

### gnomAD is (not) without error

The Genome Aggregation Database (aka gnomAD) serves to aggregate and harmonize exome and genome sequence data from variants in large-scale sequence projects. This category is unique relative to the Dynamic Databases taxon due to its ubiquity, as it is a primary source of population frequency data. The category of gnomAD is (Not) Without Error comprises inconsistencies within the referenced population database gnomAD relative to other genetic resources. This category highlights the need for an objective review of the population frequencies even from highly referenced routinely accessed sources.

### Unverifiable Reference

The Unverifiable Reference category is defined as a cited reference or statement that does not support the assertion or findings as reported in a database and or publication, bibliographic information for the reference used to support the finding of the publication that is not listed or a cited reference within the publication not readily accessible or retrievable. Examples of such discrepancies were evident in articles dating from 2013 and as recent as 2018. Unverifiable references are not restricted to journal peer-reviewed publications and may involve online resources. This type of discrepancy is classified as an Unverifiable Reference as ClinVar entries serve as a referenced source both within the database and within the published medical literature. An Unverifiable Reference is disconcerting as clinical genetic professionals depend on peer-review publications and genetic recourses for information to help provide the context necessary for classification and subsequent interpretation. Given their very existence, one may reasonably assume that these peer-reviewed published articles and professionally recommended databases have been properly curated. However, this category of potential pitfalls suggests that the referenced information may be difficult to verify and may not be verifiable at all. Similar to the consequence of other types of discrepancies discussed herein, an Unverifiable Reference may lead to misrepresentation, devaluation, or dismissal of the source article’s findings, and such erroneous information could persist and or could be perpetuated.

### Imperfect *in Silico*

The Imperfect *in Silico* category is defined as discrepant findings in public or private online computer programs or data sets that are used for predictions/modeling. Different *in silico* predictors may produce widely divergent results relative to each other. For this reason, *in silico* predictors are amongst the weakest ACMG/AMP evidence category for classifying pathogenic and likely pathogenic variants [1]. This again highlights the necessity for the routine utilization of more than one type of *in silico* tool in an analysis.

### Dynamic Databases

A Dynamic Database can be defined as an inconsistency found within the public and or commercial variant databases. These databases serve as supporting resources for curated information ideal for rapidly identifying reported accounts or publications involving the variant.

### Lack of Controls

A Lack of Controls can be defined as a failure to use controls and or population databases, inadequate controls, misinterpreted population frequency, or the use of ethnically unmatched controls. There were frequent examples identified where a variant was described as rare when population frequency data suggested otherwise. This category highlights the need for cross-referencing variant frequencies or assertions of rareness presented in studies and with large population databases.

### Facts vs. Figures

The category of Facts vs. Figures is defined as an incidence where the text and images concerning the variant in question are inconsistent with each other and/or do not support the finding(s). A Fact vs. Figure pitfall is particularly disconcerting compared to the Presumed Typos as it can involve a discrepancy that may go unnoticed and or cannot be readily resolved without clarification from the author(s).

Similar to a Presumed Typo, a Fact vs Figure discrepancy may lead to failure of the variant and related information being found in an article or may result in misrepresentation, devaluation, or dismissal of the article’s findings. Additionally, the erroneous information could be perpetuated.

### Critical Judgment

The category of Critical Judgment is defined as a failure of the researcher(s) to recognize an inconsistency that undermines the assertion of a variant’s degree of pathogenicity. This type of discrepancy can be considered complex as the recognition and resolution of it require a certain level of competence and experience in clinical genetics. Critical Judgment discrepancies highlight the potential benefits of curated databases such as HGMD Pro® which includes limited interpretive comments on select articles. It also highlights the potential shortfalls of programs that list or extract data from publications without any interpretive or summary information such as Mastermind®.

### Duplicate Counts

Duplicate Counts can be defined as a situation in which the identical or seemingly identical patient or case reports appear in more than one published study and thereby risk overrepresentation.

### Overreached & Overrepresented

The category of Overreached & Overrepresented is defined as a publication or evidence that appears in an abundance of entries within databases or scientific literature. The examples (see S1 Dataset) provide evidence that a single publication may have a disproportionate impact on clinical variant interpretation and may be misleading.

### Compound Pitfalls

A Compound Pitfall is defined as a grouping of categories described herein. This category is particularly insidious in that it requires a recognition of more than one of the types of discrepancies previously described or novel types that have yet to be described.

### Unspecified/uncharacterized/uncategorized other

There are likely existing possible pitfalls in variant analysis that have not as yet been identified. New entries and publications, version updates, and novel resources present an opportunity for new forms or types of discrepancies to appear. Therefore, this unofficial category serves as a placeholder for other types of discrepancies to be stored, described, later analyzed, and then named.

## Discussion

This study provides real-world data entailing a wide range of discrepancy types found within public and professional genomic resources used for clinical variant analysis including peer-reviewed literature and curated academic sources. Collectively, the discrepancies can be classified into eleven categories and structured as a faceted taxonomy. These categories—individually or combinations thereof—may influence the analysis and interpretation of clinically relevant variants and may contribute to the phenomenon of conflicting variant assertions of clinical significance by clinical laboratories. The types of errors suggest that they can be challenging to identify and may require dedicated resources to address and resolve them. The taxonomic model and modified schema of human errors as applied to variant analysis suggest that identified genomic datum requires routine verification—regardless of the source—by cross-referencing with overlapping or redundant resources and tools. Moreover, this study provides ancillary evidence for widening the scope of the institutional training of variant analysis as a multidisciplinary subject for both academic and clinical genetic professionals. Further research of this topic may assist in identifying additional discrepancy categories and may enable quantification estimates of the discrepancies in an effort to minimize the potential impact. Additionally, the taxonomic model suggests that a standardized algorithm for variant analysis could shift the purposed facet taxonomic form into a networked taxonomy and thereby potentially reduce conflicting variant assertions.

There is no singular standard “one size fits all approach” to the process of variant analysis and the subsequent classification of clinical significance. Variant analysis is relatively dynamic and subject to elements of the individual algorithms employed for analysis of the specific variant, gene, and disease. Additionally, it is influenced by the selected resources and available tools, the analyst’s interpretation, the patient’s/subject’s history, the guidelines of the institution, and the relationships and conditions encompassing these factors. Indeed, as is the case, a discrepancy cannot be identified without a relative association between the analyst, the resources, and the tools used for the variant analysis.

While ClinGen is developing gene and/or disease-specific guidelines to employ in clinical variant analysis and interpretation, the process is still young. Considering the large number of known genes alone, the ClinGene resources may remain relatively modest for the foreseeable future. As a consequence, discrepant data and assertions due to the lack of variant analysis standards should be expected to persist in both research findings and clinical results.

There are existing taxonomic models customarily used in organizing data. A flat taxonomy consists of a list of categories. Hierarchical taxonomies are arranged in order of importance and are best suited when relationships and features are known. A networked taxonomy contains elements of hierarchical and associative polytaxonomic models [9]. The evidence from this study demonstrates that there can be single classifications as well as associations between the identified categories, without hierarchies. Taken as a whole, this study’s taxonomy depicts non-competing classes of errors identified in medical literature and databases. Thus, the taxonomic schema of the discrepancies, as presented herein, is necessarily a faceted taxonomic model. Future qualitative and or quantitative research into this topic may indicate an alternative taxonomic schema may be applicable, if not more appropriate.

Derived from the French word *facette* meaning small face, a faceted taxonomy allows many perspectives (or faces) [9]. This taxonomic model does not require complete knowledge of the entities or their relationships. A faceted taxonomy can be flexible allowing multiple classifications. However, limitations to this type of model include difficulty in identifying and selecting the appropriate facets, expressing relationships between elements, and creating a detailed visual representation. Adjusted temporally and spatially, the faceted taxonomic model proposed can accommodate forms that resemble a dynamic version of Reason’s Swiss cheese model [16].

Reason’s Swiss cheese model is a widespread model used for analyzing medical errors and patient safety incidents. Reason proposed a metaphor of "Swiss cheese" to explain the occurrence of system failures, such as medical mishaps [17] According to this model, in a complex system, hazards are prevented from causing human losses by a series of barriers. Each barrier has unintended weaknesses or holes. These weaknesses are inconsistent–i.e., the holes open and close at random. When by chance all holes are aligned, the hazard reaches the patient and causes harm. This model draws attention to a system, as opposed to the individual, and to randomness, as opposed to deliberate action, in the occurrence of medical errors [17].

**Fig 1:**
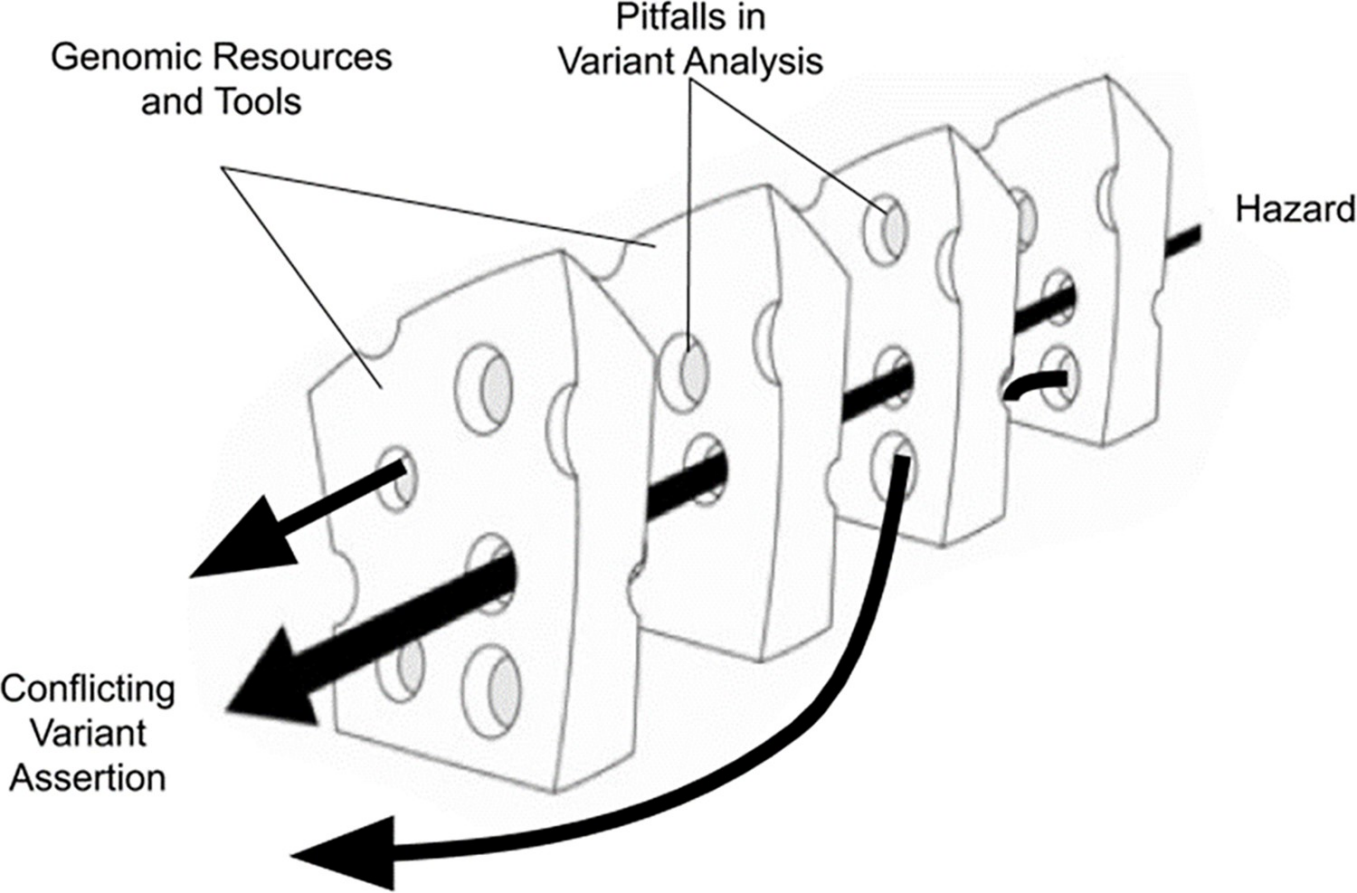
Adaptation of Reason’s Swiss cheese model. A Modified schematic model of the Reason’s Swiss cheese model of successive layers of defense barrier and safeguards as applied to variant analysis and interpretation.

In this modified and expanded model, the barriers (or the slices) are distinct types of information derived from genomic resources and tools used for variant analysis. The weaknesses (the holes) are the distinct type of pitfalls that may occur. While the appearance of the holes may be random, consistent with a lack of standardization, and the rapidly evolving nature of genetics, the model is temporally and spatially dynamic, with both the barriers and pitfalls moving in multiple dimensions and in no order at any given time. As illustrated by the black arrows (Fig 1), the hazard’s potential path (or trajectory) may lead directly to a variant assertion, may be permitted to travel through another barrier, and or may be compounded by additional pitfalls in other barriers.

This model also accommodates for the same type of pitfall in the same barrier as an individual hole may vary in size; the hole size being proportionate to the relative significance of the pitfall(s) or combination thereof. Of particular importance, the model suggests that the root cause of discrepancies may be difficult to ascertain due to the multi-dynamic elements and or the total number of elements.

Alternatively, a network taxonomy organizes content into both hierarchical and associative categories where categories can be linked to any other categories, and relationships among items can have different meanings. Hypothetically, a shift towards a network taxonomic structure of pitfalls might lower the multi-dynamic nature of this modified schema. Such a taxonomic model could be achieved by the standardization of resources and approaches.

This study and the dataset used were designed to be exploratory and, due to the dynamism of genetic resources, are stochastic in nature. The study was not designed to be systematic, comprehensive, or quantitative. Different disease or variant types may yield other findings. The dataset and method utilized a manual method of variant analysis and findings identified at the time of the analysis are presented here. It is important to note that the sources and resources for data are continually evolving, improving, and adding and deleting entries. They may also become commercialized at any time, thus removing them from free public use. In addition, advances in AI and automated programs may produce different results and may incorporate cross-referencing features and or alerts of possible discrepancies. Automated analytical pipelines may yield different findings and taxonomic models. Technical pitfalls associated with variant calling are beyond the scope of this study. Finally, categories that could be extrapolated from the ACMG/AMP guidelines and recommendations have not been incorporated into this model.

## Supporting information

S1 Dataset A categorized list of discrepancies. (DOC)

S1 Appendix References associated with the S1 Dataset and Table 1. (DOCX)

## References

[1] Richards S, Aziz N, Bale S, Bick D, Das S, Gastier-Foster J, et al. Standards and guidelines for the interpretation of sequence variants: a joint consensus recommendation of the American College of Medical Genetics and Genomics and the Association for Molecular Pathology. Genet Med. 2015;17(5):405–24. doi: 10.1038/gim.2015.30 25741868 PMC4544753

[2] College of American Pathologists, Supplemental Guide, Molecular Genetic Pathology Checklist, https://www.acgme.org/globalassets/pdfs/milestones/moleculargeneticpathologysupplementalguide.pdf Accessed February 12, 2023.

[3] Miner ClinVar, Variants with conflicting interpretations, by significance. https://clinvarminer.genetics.utah.edu/variants-in-conflict-by-significance. Accessed January 12, 2023.

[4] Xiang J, Yang J, Chen L, et al. Reinterpretation of common pathogenic variants in ClinVar revealed a high proportion of downgrades. Sci Rep. 2020;10(1):331. doi: 10.1038/s41598-019-57335-5 31942019 PMC6962394

[5] Makady A, Boer A de, Hillege H, Klungel O, Goettsch W. What is real-world data? A review of definitions based on literature and stakeholder interviews. Value in Health. 2017;20(7):858–865. doi: 10.1016/j.jval.2017.03.008 28712614

[6] Averitt AJ, Weng C, Ryan P, Perotte A. Translating evidence into practice: eligibility criteria fail to eliminate clinically significant differences between real-world and study populations. NPJ Digit Med. 2020;3:67. doi: 10.1038/s41746-020-0277-8 32411828 PMC7214444

[7] Blonde L, Khunti K, Harris SB, Meizinger C, Skolnik NS. Interpretation and impact of real-world clinical data for the practicing clinician. Adv Ther. 2018;35(11):1763–1774. doi: 10.1007/s12325-018-0805-y 30357570 PMC6223979

[8] Gilchrist A. Taxonomies and information architecture. Scire. 2003 Jun; 9(1): 37–46.

[9] Hedden H. The Accidental Taxonomist. Second edition. Medford, New Jersey: Information Today, Inc; 2016.

[10] “taxonomy, n” Oxford English Dictionary online. Oxford University Press, December 2022. Accessed 22 February 2023.

[11] Columbia Public Health, Content analysis method and examples, https://www.publichealth.columbia.edu/research/population-health-methods/content-analysis#:~:text=Courses-,Overview,words%2C%20themes%2C%20or%20concepts. Accessed February 13, 2023.

[12] Elo S, Kyngäs H. The qualitative content analysis process. Journal of Advanced Nursing. 2008;62(1):107–115. doi: 10.1111/j.1365-2648.2007.04569.x 18352969

[13] Graneheim UH, Lindgren B-M, Lundman B. Methodological challenges in qualitative content analysis: A discussion paper. Nurse Education Today. 2017;56:29–34. doi: 10.1016/j.nedt.2017.06.002 28651100

[14] Hsieh H-F, Shannon SE. Three approaches to qualitative content analysis. Qual Health Res. 2005;15(9):1277–1288. doi: 10.1177/1049732305276687 16204405

[15] “pitfall”, n.”. Oxford English Dictionary online. Oxford University Press, December 2022. Accessed 22 February 2023.

[16] Reason J. Human error: models and management. BMJ. 2000;320(7237):768–770. doi: 10.1136/bmj.320.7237.768 10720363 PMC1117770

[17] Perneger TV. The Swiss cheese model of safety incidents: are there holes in the metaphor? BMC Health Services Research. 2005;5(1):71. doi: 10.1186/1472-6963-5-71 16280077 PMC1298298

