# Supplementary material for: A taxonomic schema of potential pitfalls in clinical variant analysis based on real-world evidence
Source: PLoS One. 2023 Nov 30;18(11):e0295010. doi: 10.1371/journal.pone.0295010 (PMC10688707; doi:10.1371/journal.pone.0295010)
Supplement: S1 Dataset — (DOC) [file pone.0295010.s001.doc]

S1 Dataset. A categorized list of discrepancies.

**The Presumed Typo**

1. NM_000481.2(ATM) (no specific variant; gene only)
   [Swanson et al., 2015](https://pubmed.ncbi.nlm.nih.gov/26179960/) lists an ATM variant as “c.344+2+2insT” in the Supplementary Information Table 1. Note that this nomenclature greatly deviates from both legacy and HGVS nomenclature standards.
2. *NM_000458.2(HNF1B):c.1144C>T (p.Gln382*)

The variant protein is incorrectly listed with a superscript “c” (as opposed to the use of an asterisk or the legacy “X” for ter) in Table 2 of the article by [Madariage et al., 2018](https://pubmed.ncbi.nlm.nih.gov/31198537/).

1. NM_000016.4(ACADM):c.753-6C>A
   The variant is incorrectly listed as “c.753(-6) C>A” in Table 3 of [Waisbren et al., 2013](https://pubmed.ncbi.nlm.nih.gov/23798014/).
2. NM_000152.3(GAA):c.1316T>A (p.M439K)

The variant was incorrectly described with regards to its functional impact in the text of [Hyung-Doo Park et al., 2013](https://pubmed.ncbi.nlm.nih.gov/23884227/) as “Two mutations, c.1316T>A (p.M439K) and c.1316T>A (p.M439K), comprised 23.3% (7/30 allele) and 13.3% (4/30 alleles,) of the total mutant alleles found, respectively .”

1. NM_003742.2(ABCB11):c.2783_2787dupGAGAT (p.Lys930Glufs*79)

The variant is described as a “Patient 6 has compound heterozygous homozygous mutations” in the text of the [Siebold et al., 2013](https://pubmed.ncbi.nlm.nih.gov/20583290/). Table 1 suggests the variant is a compound heterozygous.

1. NM_000169.3(GLA):c.937G>T (p.D313Y)
   This missense variant that was described as a “nonsense” variant in the text of [Biagini et al., 2017](https://pubmed.ncbi.nlm.nih.gov/29044343/).
2. NM_000169.2(GLA):c.547G>A (p.Gly183Ser)

This missense variant is incorrectly described as a deletion in [Tuttolomondo et al., 2017](https://pubmed.ncbi.nlm.nih.gov/28977874/).

1. NM_000443.3(ABCB4):c.857C>T (A286V)

This variant was described as incorrectly as “Ala287Val” in Table 1 of [Poupon et al., 2010](https://pubmed.ncbi.nlm.nih.gov/20537830/). Interestingly, the variant is correctly listed as p.Ala286Val” in Table 2 [Wendum et al., 2012](https://pubmed.ncbi.nlm.nih.gov/22331132/) – an article that has three common authors to Poupon et al. There are no known alternative transcripts that have adjacent alanines in either one of these position.

1. NM_001353921.2(ARHGEF9):c.865C>T (p.Arg289*)
   The genomic chromosome and coordinates of this variant is incorrectly listed in the Supplemental Table of [Retterer et al., 2016](https://pubmed.ncbi.nlm.nih.gov/26633542/). The correct position is listed in the previous two rows. A cursory review of the coordinates of the other variants listed in this supplemental suggest that other variants may have been listed with the incorrect chromosomal coordinates listed.
2. NM_000095.2(COMP):c.1467C>A (p.Asn489Lys)
   This variant was described as a different base change. [Briggs et al., 2002](https://pubmed.ncbi.nlm.nih.gov/11968079/) described the variant in Table 2 incorrectly as “1467G>C” whereas [Briggs et al., 2014](https://pubmed.ncbi.nlm.nih.gov/24595329/) describe the variant as “c.1467C>A” in Table 1. The primary authors of the two article are the same.
3. NM_017780.3 (CHD7):c.5405-17G>A

Described in the text as a “splice site mutation”, this variant was classified as being both “Pathogenic” and “VUS” using “ACMG variant classification standards” as reported in Table S7 of [Aref-Eshghi et al., 2018](https://pubmed.ncbi.nlm.nih.gov/29304373/). The paper lists the variant 3 times in reference to 3 different patient IDs.

1. NM_000392.5(ABCC2):c.2443C>T (p.R815*)

The variant is incorrectly reported as “p.R815a” in Table S2 of [Wang et al., 2020](https://pubmed.ncbi.nlm.nih.gov/31450232/). At this time of writing, this article is the only known account of any patient with this variant.

1. NM_006005.3(WFS1):c.2486T>C (p.L829P)

Variant is incorrectly described as “c.2656T>C” in the text of [Besplaova et al., 2001](https://pubmed.ncbi.nlm.nih.gov/11709537/). The cDNA number is incorrect. This cDNA numbering is inconsistent with known transcripts and versions.

1. CFTR:Ex13:c.1680-886A>G (aka 1811+1.6kbA>G) This is an example of an unverifiable reference that overlaps with a presumed typo. The variant is described in Table 4 of [Castellani et al., 2008](https://pubmed.ncbi.nlm.nih.gov/18456578/). Herein, reference #153 is provided as supporting evidence of the variant’s pathogenicity, however, reference #153, Highsmith, et al. (1997), does not contain the variant. Upon further inspection, however, reference #154, Chillón, et al.(1995), is the appropriate reference and we can presume this was a typo.

**gnomAD is (Not) Without Error**

1. rs1388716999
   Variant [rs1388716999](https://www.ncbi.nlm.nih.gov/snp/rs1388716999) (a single base pair deletion per dbSNP) is identified in [gnomAD](https://gnomad.broadinstitute.org/variant/17-19555854-A-G?dataset=gnomad_r2_1) as [rs763186740](https://www.ncbi.nlm.nih.gov/snp/rs763186740) (a missense per dbSNP).
2. rs1554331549
   Variant [rs1554331549](https://www.ncbi.nlm.nih.gov/snp/rs1554331549) (a missense per dbSNP) is identified in [gnomAD](https://gnomad.broadinstitute.org/variant/17-19555854-A-G?dataset=gnomad_r2_1) as [rs779852596](https://www.ncbi.nlm.nih.gov/snp/rs779852596) (a 3 base pair deletion per dbSNP).
3. rs398123301
   Variant [rs398123301](https://www.ncbi.nlm.nih.gov/snp/rs398123301) (a deletion per dbSNP) is identified in [gnomAD](https://gnomad.broadinstitute.org/) as [rs768427035](https://www.ncbi.nlm.nih.gov/snp/rs768427035) (a missense per dbSNP).
4. rs750693623
   Variant [rs750693623](https://www.ncbi.nlm.nih.gov/snp/rs750693623) (a deletion per dbSNP) is identified in gnomAD as [rs1465540247](https://www.ncbi.nlm.nih.gov/snp/rs1465540247) (a missense per dbSNP).
5. rs985355103
   Variant rs985355103 is listed as a large multi-base deletion (in all associated transcripts) in [gnomAD](https://gnomad.broadinstitute.org/variant/9-71836045-GAGCCGTGGCCGGAGCCTGGAGCGGGGCCTGGACCAAGACCATGCGCGCACCCGAGACCGC-G?dataset=gnomad_r2_1). However, [dbSNP](https://www.ncbi.nlm.nih.gov/snp/rs985355103) lists the variant as a substitution (in all transcripts listed).
6. rs147748659
   Variant rs147748659 is listed in [gnomAD](https://gnomad.broadinstitute.org/variant/10-100183511-G-T?dataset=gnomad_r2_1) as “a splice region” in the 6 transcripts of this gene. However, [dbSNP](https://www.ncbi.nlm.nih.gov/snp/rs147748659) lists the variant as a synonymous/missense variant in all transcripts of the gene.
7. rs797045295
   This X linked variant was listed in [gnomAD](https://gnomad.broadinstitute.org/variant/X-25031809-T-C) with a total allele count of 2 however, there were not homozygous or hemizygotes listed. Note: This variant entry has been updated to reflect that it is found in a “low complexity region”. This note did not appear at the initial time of inquiry.

**The Unverifiable Reference**

1. NM_022067.3(VIPAS39):c.484C>T (p.R162*)

[Zohou et al., 2014](https://pubmed.ncbi.nlm.nih.gov/25239142/) references article #14 in Table 3 in support of the pathogenic classification of the variant, however, the [referenced article](https://pubmed.ncbi.nlm.nih.gov/21922076/) does not contain the variant.

1. NM_022437.2(ABCG8):c.584T>A (p.Arg184His)

[Andolfo et al., 2018](https://pubmed.ncbi.nlm.nih.gov/28971506/) reference article #129 in Table 1 in support of the variant, however there is no reference #129 listed at the end of the article.

1. NM_001360.2(DHCR7):c.461C>G (T154R)

[Jira et al., 2003](https://pubmed.ncbi.nlm.nih.gov/12914579/) references article #8 (DeBrasi et al, 1999) in Table 2 in support of the pathogenic classification of the variant, however, the referenced article does not contain the variant. Interestingly, Jira et al., 2003 does not cite the T93M variant in their paper Table 2 which is prominently depicted in the Debrasi et al, 1999 article.

1. NM_001360.2(DHCR7):c.461C>G (T154R)
   A large reference clinical laboratory cited “[PMID: 20694756](https://pubmed.ncbi.nlm.nih.gov/20694756/)” in the “Evidence details” column of the ClinVar entry [VCV000166988.8](https://www.ncbi.nlm.nih.gov/clinvar/variation/166988/). However, the variant is not described in detail in the abstract.
2. NM_000235.2(LIPA):c.929G>A (p.Trp310*)

The HGMD® database entry (CM1620196) lists a reference that could not be found in Google Scholar, Pubmed nor the Clemson Library. There was only a single reference listed (Elsayed (2016). Egypt J Med Human Genet, 277).

1. NM_000169.2(GLA):c.-10C>T

The HGMD® Pro database entry (CR088476) lists an article “Desnick (2015) Mol Genet Meta 114:S37” which could not be found using PubMed nor Google Scholar. This was article was a conference abstract published in the journal and the only functional paper listed, *Fabry disease: The α-galactosidase A (GLA) c.427GNA (A143T) mutation, effect of the 5′-10CNT polymorphism*. Comments by HGMD regarding this were “15-20% lower activity than WT”. From the abstract, the section read, “10T had about 15-20% lower luciferase activity than that with the more common −10C wild-type sequence”

1. NM_000350.2(ABCA4):c.4297G>A (p.V1433I)

At the time of this review, a variant was listed as pathogenic in The Leiden Open Variation Database (LOVD); with a reference to [Koyanagi et al., 2019](https://pubmed.ncbi.nlm.nih.gov/31213501/). The article stated that “All data relevant to the study in the article or upload as supplementary information.” The supplemental is not readily available as the website ([The Japanese Retinitis Pigmentosa Registry Project](https://secure2.visitors.jp/retinal_pigment/login/)) is under construction, password protected with instructions in Japanese.

**Imperfect in Silico**

1. NM_004817.3(TJP2):c.2335G>A (p.Val779Met) (aka chr9[hg19]:g.71853685)

The variant is purported to be towards pathogenic along the spectrum of being benign to pathogenic by the in silico program [CADD](https://cadd.gs.washington.edu/snv). The variant’s CADD score is 28.9 (with a range of 0-32, with the value of 32 being the most detrimental prediction). The variant is purported to be towards benign in [Revel](https://sites.google.com/site/revelgenomics/downloads). The Revel score of 0.303 (with a range of 0-1 with 1 being the most detrimental prediction).

1. NM_023073.3(CPLANE1):c.2624C>T (p.Ser875Phe)
   The variant is not recognized its HGVS gene name in the [Human Splicing Finder](http://umd.be/Redirect.html) (GNEOMIS) in silico program. The legacy gene name of this variant, “C5ORF42” is recognized by the program.
2. NM_001080420.1(SHANK3):C.4526_4527delTG – Per current NCBI reference, this Reference Sequence has been permanently suppressed due to insufficient support for the transcript and the protein. Exon 11 was based on ab initio prediction and is not supported by transcript data. At the time of this review, the “NM_001080420.1:c.4526_4527delTG” variant was inputted into the [Mutalyzer’s Position Converter](https://mutalyzer.nl/position-converter?assembly_name_or_alias=GRCh37&description=NM_001080420.1%3Ac.4526_4527delTG) with results in “NM_033517.1:c.441_442delTG”. However, using the “NM_033517.1:c.441_442delTG” as input in the [Mutalyzer’s Name Checker](https://mutalyzer.nl/name-checker?description=NM_033517.1%3Ac.441_442delTG) results in an error “TG not found at position 441_442, found CG instead.”
3. NM_003742.2 (ABCB11):c.1774G>T (p.E592*)
   [Mutalyzer](https://mutalyzer.nl/name-checker?description=NM_003742.2:c.1774G>T) incorrectly states that the variant is located in the one and only exon of this gene. However, this gene has 30 exons per the [NCBI Gene](https://www.ncbi.nlm.nih.gov/gene/8647) database.
4. No Specific Variant
   Revel is a freely accessible in silico program used for predicting the impact of a variant on the protein. Predetermined values are downloaded from their website. In this example, the coordinates were found to be erroneous in that the predetermined values for the X and Y chromosomes were incorrectly listed as being in Chromosomes 9. (Downloads)
5. NM_000029.4:c.803T>C (p.Met268Thr) (aka rs699)
   The variant was one of many used to identify an error in which [Ensemble Genome Browser](https://www.ensembl.org/Homo_sapiens/Variation/Population?db=core;r=1:230709548-230710548;v=rs699;vdb=variation;vf=179) failed to display 1000 Genomes frequency data. The error was restricted to servers dedicated to queries originating from the United States. Correspondence with the help desk for ensemble.org (2020) indicated they were working toward restoring U.S. mirrors.
6. No Specific Variant
   Once freely available, [Human Splicing Finder (HSF)](http://umd.be/Redirect.html) and a recommended ClinGen tool was restricted for commercial use in January 2020. This was also a previously ACMG/AMP recommended tool (Richards, et al., 2015).

**Dynamic Databases**

1. NM_138735.4(NRXN1):c.41C>T (p.S14)
   This variant is listed among other variants in commercial HMGD Pro 2020.2 and [the public version](http://www.hgmd.cf.ac.uk/ac/gene.php?gene=nrxn1b) of the database under the gene *NRXN1b*. There is no human gene named “*NRXN1b*” nor is it a legacy name, as confirmed by the widely used reference databases [OMIM](https://omim.org/search?index=entry&start=1&limit=10&sort=score+desc%2C+prefix_sort+desc&search=nrxn1b) and [NCBI Gene](https://www.ncbi.nlm.nih.gov/gene/?term=nrxn1b).
2. NM_000137.2(FAH):c.836A>T (p.Q279L)

The variant had two district entries in the HGMD database (CM005583 and CS015389) at the time of this exploration. The variant was listed as a compound heterozygote splice site and missense variants causing disease. The literature citations differ in the number of citations for each entry. Entry CM005583 has 5 citations whereas entry CS015389 has 2 citations. Moreover, one of the citations in CS015389 was unique to this entry.

1. NM_138694.3(PKHD1):c.8492G>A (p.A2831K) **Quagga-check Qgene pro database.**

The variant is listed as “Does not affect function” in the [LOVD](https://databases.lovd.nl/shared/variants/0000087697#00000115) database (Genomic Variant #0000087697) which had [Bergmann et al., 2005](https://pubmed.ncbi.nlm.nih.gov/15698423/) listed as a supporting reference which did not state that the variant was benign. The variant is listed as a “DM” in the HGMD database entry CM051168 with Bergmann et al., 2005 as the sole supporting literature citation.

**Lack of Control(s)**

1. NM_005603.4(ATP8B1):c.134A>C (p.Asn45Thr)
   This variant is described as “rare” in [Kulecka et al., 2017](https://pubmed.ncbi.nlm.nih.gov/28626473/). However, the [gnomAD](https://gnomad.broadinstitute.org/variant/18-55398906-T-G) entry indicates that the variant had a frequency of 0.5% in the general population with 6 ostensibly healthy homozygotes being reported.
2. NM_000458.3(HNF1B):c.*99C>A (aka rs2229295)
   This variant was described as “significantly associated” with type 2 diabetes ([Goda et al., 2015](https://www.ncbi.nlm.nih.gov/pmc/articles/PMC4557749/)). Laboratories (2) submitting to ClinVar suggests this variant is benign though one lab cited the above article. The [gnomAD](https://gnomad.broadinstitute.org/variant/17-36047276-G-T?dataset=gnomad_r2_1) reported the allele frequency to be greater than ~50% in the African population and greater than 22% in the Ashkenazi Jewish population.
3. NM_001126131.2(POLG):c.2890C>T (p.Arg964Cys) rs201477273

This variant was characterized in a recent Taiwanese population study by [Hsiech et al., 2019](https://pubmed.ncbi.nlm.nih.gov/30941926/) as being “likely pathogenic” per ACMG guidelines. The researcher’s stated “We considered variants with minor allele frequency of </=0.1% (rare variants)”. Moreover, the researcher’s stated that variant was absent from controls or had extremely low frequency in the Exome Sequencing Project, 1000 Genomes or ExAC. In fact, the variant has been reported at frequency as high as 0.9% in the East Asian subpopulation in [gnomAD](https://gnomad.broadinstitute.org/variant/15-89864088-G-A?dataset=gnomad_r2_1) .

1. ALDH3A2, NM_000382.2(ALDH2A2):c.386-6A>G (rs117330764)

This variant is described as rare in a study by [Amr et al., 2019](https://pubmed.ncbi.nlm.nih.gov/31388754/) on account that the variant was not found in “100 normal chromosomes of Egyptian origin”. In fact, the variant can be considered common per ACMG/AMP recommendations as it has been reported as having a frequency of ~5.3% in the African population per [gnomAD](https://gnomad.broadinstitute.org/variant/17-19555854-A-G?dataset=gnomad_r2_1) data. It was found in 42 ostensibly healthy homozygotes in the general population.

**Facts vs. Figures**

1. NM_003482.3(KMT2D):c.13259G>A (p.Arg4420Gln)
   The variant which is stated as a novel missense mutation in [Cheon et al., 2014](https://pubmed.ncbi.nlm.nih.gov/24739679/) is inconsistent with the electropherogram which depicts a frameshift that is not described in the article.
2. NM_000275.2(OCA2):c.593C>T (p.Pro198Leu)
   This variant is described in the text and Table 1 of [Yang et al., 2019](https://pubmed.ncbi.nlm.nih.gov/31196117/) as being homozygous in the proband. The father and mother are listed as a carrier for the variant. However, the electropherogram of Patient 9 indicates the proband is heterozygous. This example could be classified as a presumed typo – as the labeling of the electropherogram may have simply been labeled incorrect. However, because this example involves a figure as evidence of the variant’s presence in the proband, it is categorized as Fact vs. Figure.
3. NM_000481.4(AMT):c.136G>A (p.Gly46Ser)
   The variant which described in Table 3 of [Kure et al., 2006](https://pubmed.ncbi.nlm.nih.gov/16450403/) incorrectly describes the variant as “p.G47R” and novel. Note that both the predicted amino acid and amino acids number are incorrect. There is no obvious evidence that this incident is a presumed typo.

**Critical Judgment**

1. NM_002335.3(LRP5):c.1828G>T (p.G610W)
   The variant was described in [Xia et al., 2017](https://pubmed.ncbi.nlm.nih.gov/28677207/) as homozygous in non-twin siblings but not found in the parents. It is noted that the authors of non-English speaking origin and therefore this may be a presumed typo on account of being lost in translation as opposed to an error of critical judgment.
2. NM_005379.3(MYO1A):c.2468T>C (p.Leu823Pro)
   The variant is described as de novo in a familial case in the study by [Morgan et al., 2018](https://pubmed.ncbi.nlm.nih.gov/30622556/), published online 21, December 2018. Additionally, the claim that the gene is involved in hearing impairment has been classified as “Refuted” since 1, January, 2018 by [ClinGen](https://search.clinicalgenome.org/kb/gene-validity/3dcc24f7-4cf7-413b-a956-16ad58bcbd36--2018-01-16T17:00:00).

**Duplicate Counts**

1. NM_01884803(MKKS):c.830T>C, p.L277P
   In the ClinVar entry [VCV000005314.4](https://www.ncbi.nlm.nih.gov/clinvar/variation/5314/), a prominent laboratory (GeneDx) asserted that the variant was found in two unrelated individuals with supporting publications from [Katsanis et al., 2000](https://pubmed.ncbi.nlm.nih.gov/10973251/) and [Moore et al., 2005](https://pubmed.ncbi.nlm.nih.gov/15637713/). Both publications (which share authors) describe the individual being from Newfoundland with F94fsX103/L277P alleles. It is likely that the individual is the same person in both papers.

**Overreached and Overrepresented**

1. No Specific Variant
   [Xiong et al., 2015](https://pubmed.ncbi.nlm.nih.gov/25525159/) provided in silico splicing predictions for 650,000 variants. As a result, this paper has been associated with over 17,300 entries in the HGMD Pro database. This article has been cited at least 1245 per Google Scholar and over 2000 times as supporting evidence by ClinVar reporting laboratories.

**Compound Pitfall**

1. ABCB11c.1442T>A (p.V481E)
   The variant is described in the text of [Bryne et al., 2009](https://pubmed.ncbi.nlm.nih.gov/19101985/) as a cryptic splice site mutation. However, Figure 2 is not consistent with this conclusion. The threshold used by the authors for the interpretation of this variant suggests that most of the variants are also cryptic splice sites. The paper is associated with 68 variant entries in HGMD, has been cited 169 times per Google Scholar and has been used as supporting evidence in at least 25 ClinVar variant entries. Both classification categories of Critical Judgment and Overreaching and Overrepresented apply to this example.
