# Supplementary material for: A taxonomic schema of potential pitfalls in clinical variant analysis based on real-world evidence
Source: PLoS One. 2023 Nov 30;18(11):e0295010. doi: 10.1371/journal.pone.0295010 (PMC10688707; doi:10.1371/journal.pone.0295010)
Supplement: S1 Appendix — (DOCX) [file pone.0295010.s002.docx]

S1 Appendix. References associated with the S1 Dataset.

1. ABCB11 ATP binding cassette subfamily B member 11 [Homo sapiens (Human)]—Gene—NCBI. (n.d.). Retrieved September 9, 2020, from <https://www.ncbi.nlm.nih.gov/gene/8647>
2. Abstracts of the Annual Symposium of the Society for the Study of Inborn Errors of Metabolism. Istanbul, Turkey. August 31-September 3, 2010. (2010). Journal of inherited metabolic disease, 33 Suppl 1, S20–S187. <https://doi.org/10.1007/s10545-010-9163-x>
3. Adam MP, Ardinger HH, Pagon RA, et al., editors. GeneReviews® [Internet]. Seattle (WA): University of Washington, Seattle; 1993-2020. Available from: <https://www.ncbi.nlm.nih.gov/books/NBK1116/>
4. Amr, K., El-Bassyouni, H. T., Ismail, S., Youness, E., El-Daly, S. M., Ebrahim, A. Y., & El-Kamah, G. (2019). Genetic assessment of ten Egyptian patients with Sjögren-Larsson syndrome: expanding the clinical spectrum and reporting a novel ALDH3A2 mutation. Archives of dermatological research, 311(9), 721–730. <https://doi.org/10.1007/s00403-019-01953-6>
5. Andolfo, I., Russo, R., Gambale, A., & Iolascon, A. (2018). Hereditary stomatocytosis: An underdiagnosed condition. American journal of hematology, 93(1), 107–121. <https://doi.org/10.1002/ajh.24929>
6. Aref-Eshghi, E., Rodenhiser, D. I., Schenkel, L. C., Lin, H., Skinner, C., Ainsworth, P., Paré, G., Hood, R. L., Bulman, D. E., Kernohan, K. D., Care4Rare Canada Consortium, Boycott, K. M., Campeau, P. M., Schwartz, C., & Sadikovic, B. (2018). Genomic DNA Methylation Signatures Enable Concurrent Diagnosis and Clinical Genetic Variant Classification in Neurodevelopmental Syndromes. American journal of human genetics, 102(1), 156–174. <https://doi.org/10.1016/j.ajhg.2017.12.008>
7. Bespalova, I. N., Van Camp, G., Bom, S. J., Brown, D. J., Cryns, K., DeWan, A. T., Erson, A. E., Flothmann, K., Kunst, H. P., Kurnool, P., Sivakumaran, T. A., Cremers, C. W., Leal, S. M., Burmeister, M., & Lesperance, M. M. (2001). Mutations in the Wolfram syndrome 1 gene (WFS1) are a common cause of low frequency sensorineural hearing loss. Human molecular genetics, 10(22), 2501–2508. <https://doi.org/10.1093/hmg/10.22.2501>
8. Biagini, G., Almeida, A., Almeida, T., Silva, C., Castro, B. F., Reche, T. C., Dabinski, A. C., & Barreto, F. C. (2017). Case report: is low α-Gal enzyme activity sufficient to establish the diagnosis of Fabry disease? Jornal brasileiro de nefrologia : 'orgao oficial de Sociedades Brasileira e Latino-Americana de Nefrologia, 39(3), 333–336. <https://doi.org/10.5935/0101-2800.20170057>
9. Briggs, M. D., & Chapman, K. L. (2002). Pseudoachondroplasia and multiple epiphyseal dysplasia: mutation review, molecular interactions, and genotype to phenotype correlations. Human mutation, 19(5), 465–478. <https://doi.org/10.1002/humu.10066>
10. Briggs, M. D., Brock, J., Ramsden, S. C., & Bell, P. A. (2014). Genotype to phenotype correlations in cartilage oligomeric matrix protein associated chondrodysplasias. European journal of human genetics: EJHG, 22(11), 1278–1282. <https://doi.org/10.1038/ejhg.2014.30>
11. Byrne, J. A., Stautnieks, S. S., Ihrke, G., Pagani, F., Knisely, A. S., Linton, K. J., …Thompson, R. J. (2009). Missense mutations and single nucleotide poly morphisms in ABCB11 impair bile salt export pump processing and function or disrupt pre-messenger RNA splicing. Hepatology, 49(2), 553-67. doi:10.1002/hep.22683
12. CADD—Combined annotation dependent depletion. (n.d.). Retrieved September 9, 2020, from <https://cadd.gs.washington.edu/snv>. Revel website. Retrieved September 9, 2020, from https://sites.google.com/site/revelgenomics/downloads
13. Castellani, C., Cuppens, H., Macek, M., Jr, Cassiman, J. J., Kerem, E., Durie, P., Tullis, E., Assael, B. M., Bombieri, C., Brown, A., Casals, T., Claustres, M., Cutting, G. R., Dequeker, E., Dodge, J., Doull, I., Farrell, P., Ferec, C., Girodon, E., Johannesson, M., … Elborn, J. S. (2008). Consensus on the use and interpretation of cystic fibrosis mutation analysis in clinical practice. Journal of cystic fibrosis: official journal of the European Cystic Fibrosis Society, 7(3), 179–196. <https://doi.org/10.1016/j.jcf.2008.03.009>
14. Cheon, C. K., Sohn, Y. B., Ko, J. M., Lee, Y. J., Song, J. S., Moon, J. W., Yang, B. K., Ha, I. S., Bae, E. J., Jin, H. S., & Jeong, S. Y. (2014). Identification of KMT2D and KDM6A mutations by exome sequencing in Korean patients with Kabuki syndrome. Journal of human genetics, 59(6), 321–325. <https://doi.org/10.1038/jhg.2014.25>
15. Chillón, M., Dörk, T., Casals, J., Gimėnemz, Fonknechten, N., Will, K., …Nunes, V. (1995). A novel donor splice site in intron 11 of the CFTR gene, created by mutation 1811 + 1.6kbA→G, produces a new exon: High frequency in Spanish cystic fibrosis chromosomes and association with severe phenotype. American Journal of Medical Genetics, 56, 623-629.
16. DeBrasi, D., Esposito, T., Rossi, M., Parenti, G., Sperandeo, M. P., Zuppaldi, A., …Andria, G. (1999). Smith-Lemli-Opitz syndrome: evidence of T93M as a common mutation of Δ7-sterol reductase in Italy and report of three novel mutations. European journal of human genetics, 7, 937-940 **Quagga**
17. *Downloads - Revel: Rare exome variant ensemble learner. (n.d.). Retrieved September 9, 2020, from <https://sites.google.com/site/revelgenomics/downloads>
18. * Ensembel Asia. Failure to display 1000 genomes data. Retrieved September 9, 2020, from <https://asia.ensembl.org/Homo_sapiens/Variation/Population?db=core;r=1:230709548-230710548;v=rs699;vdb=variation;vf=179>
19. Genomic variant #0000087697—Global Variome shared LOVD. (n.d.). Retrieved September 8, 2020, from <https://databases.lovd.nl/shared/variants/0000087697#00000115> Bergman_Quagga
20. *Gnomad. (n.d.). HNF1B_Retrieved September 8, 2020 from

<https://gnomad.broadinstitute.org/variant/17-36047276-G-T?dataset=gnomad_r2_1>

1. *Genomad. (n.d.). POLG_Retrieved September 8, 2020 from

<https://gnomad.broadinstitute.org/variant/15-89864088-G-A?dataset=gnomad_r2_1>

1. *Genomad. (n.d.). HNF1B_Retrieved September 8, 2020 from <https://gnomad.broadinstitute.org/variant/17-36070572-T-C?dataset=gnomad_r2_1>
2. *Gnomad. (n.d.). Retrieved September 8, 2020, from https://gnomad.broadinstitute.org/variant/17-19555854-A-G?dataset=gnomad_r2_1.
3. *Gnomad. (n.d.). Retrieved September 8, 2020 from https://gnomad.broadinstitute.org/variant/18-55398906-T-G
4. *Goda, N., Murase, H., Kasezawa, N., Goda, T., & Yamakawa-Kobayashi, K. (2015). Polymorphism in microRNA-binding site in HNF1B influences the susceptibility of type 2 diabetes mellitus: a population based case-control study. BMC medical genetics, 16, 75. <https://doi.org/10.1186/s12881-015-0219-5>
5. *Halbritter, J., Porath, J. D., Diaz, K. A., Braun, D. A., Kohl, S., Chaki, M., Allen, S. J., Soliman, N. A., Hildebrandt, F., Otto, E. A., & GPN Study Group (2013). Identification of 99 novel mutations in a worldwide cohort of 1,056 patients with a nephronophthisis-related ciliopathy. Human genetics, 132(8), 865–884. <https://doi.org/10.1007/s00439-013-1297-0>
6. *HGMD® gene result. (n.d.). Retrieved September 8, 2020, from <http://www.hgmd.cf.ac.uk/ac/gene.php?gene=nrxn1b>
7. *HGMD® gene result. (n.d.). Retrieved September 8, 2020, from http://www.hgmd.cf.ac.uk/ac/gene.php?gene=WFS1
8. *HGMD® gene result. (n.d.). Retrieved September 8, 2020, from http://www.hgmd.cf.ac.uk/ac/gene.php?gene=LIPA
9. *HGMD® gene result. (n.d.). Retrieved September 8, 2020, from http://www.hgmd.cf.ac.uk/ac/gene.php?gene=GLA
10. *HGMD® gene result. (n.d.). Retrieved September 8, 2020, from http://www.hgmd.cf.ac.uk/ac/gene.php?gene=FAH
11. *Highsmith, W. E., Burch, L. H., Zhou, Z., Olsen, J.C., Strong, T. V., Smith, T., …Knoweles, M. R. (1997). Identification of a splice site mutation (2789+5G>A) associated with small amounts of normal CFTR mRNA and mild cystic fibrosis. Human Mutation, 9, 332-338.
12. *Hsieh, P. C., Wang, C. C., Tsai, C. L., Yeh, Y. M., Lee, Y. S., & Wu, Y. R. (2019). POLG R964C and GBA L444P mutations in familial Parkinson's disease: Case report and literature review. Brain and Behavior, 9(5), e01281. <https://doi.org/10.1002/brb3.1281>
13. *Human splicing finder—Version 3. 1. (n.d.). Retrieved September 9, 2020, from <http://umd.be/Redirect.html>. https://www.ncbi.nlm.nih.gov/pmc/articles/PMC4544753/
14. -Jira, P. E., Waterham, H. R., Wanders, R. J., Smeitink, J. A., Sengers, R. C., & Wevers, R. A. (2003). Smith-Lemli-Opitz syndrome and the DHCR7 gene. Annals of human genetics, 67(Pt 3), 269–280. <https://doi.org/10.1046/j.1469-1809.2003.00034.x>
15. -Katsanis, N., Beales, P. L., Woods, M. O., Lewis, R. A., Green, J. S., Parfrey, P. S., Ansley, S. J., Davidson, W. S., & Lupski, J. R. (2000). Mutations in MKKS cause obesity, retinal dystrophy and renal malformations associated with Bardet-Biedl syndrome. Nature genetics, 26(1), 67–70. <https://doi.org/10.1038/79201>
16. -Koyanagi, Y., Akiyama, M., Nishiguchi, K. M., Momozawa, Y., Kamatani, Y., Takata, S., Inai, C., Iwasaki, Y., Kumano, M., Murakami, Y., Omodaka, K., Abe, T., Komori, S., Gao, D., Hirakata, T., Kurata, K., Hosono, K., Ueno, S., Hotta, Y., Murakami, A., … Sonoda, K. H. (2019). Genetic characteristics of retinitis pigmentosa in 1204 Japanese patients. Journal of medical genetics, 56(10), 662–670. <https://doi.org/10.1136/jmedgenet-2018-105691>
17. -Kulecka, M., Habior, A., Paziewska, A., Goryca, K., Dąbrowska, M., Ambrozkiewicz, F., Walewska-Zielecka, B., Gabriel, A., Mikula, M., & Ostrowski, J. (2017). Clinical applicability of whole-exome sequencing exemplified by a study in young adults with the advanced cryptogenic cholestatic liver diseases. Gastroenterology research and practice, 2017, 4761962. <https://doi.org/10.1155/2017/4761962>
18. -Kure, S., Kato, K., Dinopoulos, A., Gail, C., DeGrauw, T. J., Christodoulou, J., Bzduch, V., Kalmanchey, R., Fekete, G., Trojovsky, A., Plecko, B., Breningstall, G., Tohyama, J., Aoki, Y., & Matsubara, Y. (2006). Comprehensive mutation analysis of GLDC, AMT, and GCSH in nonketotic hyperglycinemia. Human mutation, 27(4), 343–352. <https://doi.org/10.1002/humu.20293>
19. *Madariaga, L., García-Castaño, A., Ariceta, G., Martínez-Salazar, R., Aguayo, A., Castaño, L., & Spanish group for the study of HNF1B mutations (2018). Variable phenotype in HNF1B mutations: extrarenal manifestations distinguish affected individuals from the population with congenital anomalies of the kidney and urinary tract. Clinical kidney journal, 12(3), 373–379. <https://doi.org/10.1093/ckj/sfy102>
20. -Moore, S. J., Green, J. S., Fan, Y., Bhogal, A. K., Dicks, E., Fernandez, B. A., Stefanelli, M., Murphy, C., Cramer, B. C., Dean, J. C., Beales, P. L., Katsanis, N., Bassett, A. S., Davidson, W. S., & Parfrey, P. S. (2005). Clinical and genetic epidemiology of Bardet-Biedl syndrome in Newfoundland: a 22-year prospective, population-based, cohort study. American journal of medical genetics. Part A, 132A(4), 352–360. <https://doi.org/10.1002/ajmg.a.30406>
21. -Morgan, A., Lenarduzzi, S., Cappellani, S., Pecile, V., Morgutti, M., Orzan, E., Ghiselli, S., Ambrosetti, U., Brumat, M., Gajendrarao, P., La Bianca, M., Faletra, F., Grosso, E., Sirchia, F., Sensi, A., Graziano, C., Seri, M., Gasparini, P., & Girotto, G. (2018). Genomic Studies in a Large Cohort of Hearing Impaired Italian Patients Revealed Several New Alleles, a Rare Case of Uniparental Disomy (UPD) and the Importance to Search for Copy Number Variations. Frontiers in genetics, 9, 681. <https://doi.org/10.3389/fgene.2018.00681>
22. --Mutalyzer 2.0.32—Name Checker. (n.d.). Retrieved September 9, 2020, from <https://mutalyzer.nl/name-checker?description=NM_033517.1%3Ac.441_442delTG>
23. --Mutalyzer 2.0.32—Name Checker. (n.d.). Retrieved September 9, 2020, from <https://mutalyzer.nl/name-checker?description=NM_003742.2:c.1774G%3ET>
24. --Mutalyzer 2.0.32—Position Converter. (n.d.). Retrieved September 9, 2020, from <https://mutalyzer.nl/position-converter?assembly_name_or_alias=GRCh37&description=NM_001080420.1%3Ac.4526_4527delTG>
25. -Nrxn1b—Gene—Ncbi. (n.d.). Retrieved September 8, 2020, from <https://www.ncbi.nlm.nih.gov/gene/?term=nrxn1b>
26. -Omim entry search—Nrxn1b. (n.d.). Retrieved September 8, 2020, from <https://omim.org/search?index=entry&start=1&limit=10&sort=score+desc%2C+prefix_sort+desc&search=nrxn1b>
27. -Park, H. D., Lee, D. H., Choi, T. Y., Lee, Y. K., Lee, S. Y., Kim, J. W., Ki, C. S., & Lee, Y. W. (2013). Three patients with glycogen storage disease type II and the mutational spectrum of GAA in Korean patients. Annals of clinical and laboratory science, 43(3), 311–316.
28. --Poupon, R., Arrive, L., & Rosmorduc, O. (2010). The cholangiographic features of severe forms of ABCB4/MDR3 deficiency-associated cholangiopathy in adults. Gastroenterologie clinique et biologique, 34(6-7), 380–387. <https://doi.org/10.1016/j.gcb.2010.04.011>
29. --Retterer, K., Juusola, J., Cho, M. T., Vitazka, P., Millan, F., Gibellini, F., Vertino-Bell, A., Smaoui, N., Neidich, J., Monaghan, K. G., McKnight, D., Bai, R., Suchy, S., Friedman, B., Tahiliani, J., Pineda-Alvarez, D., Richard, G., Brandt, T., Haverfield, E., Chung, W. K., … Bale, S. (2016). Clinical application of whole-exome sequencing across clinical indications. Genet Med, 18(7), 696–704. <https://doi.org/10.1038/gim.2015.148>
30. -Richards, S., Aziz, N., Bale, S., Bick, D., Das, S., Gastier-Foster, J., …ACMG Laboratory Quality Assurance Committee. Standards and guidelines for the interpretation of sequence variants: A joint consensus recommendation of the American College of Medical Genetics and Genomics and the Association for Molecular Pathology. Genet Med, 17(5), 405-424. doi: 10.1038/gim.2015.30
31. *Rs1388716999 refsnp report—Dbsnp—Ncbi. (n.d.). Retrieved September 8, 2020, from <https://www.ncbi.nlm.nih.gov/snp/rs1388716999>
32. *Rs1465540247 refsnp report—Dbsnp—Ncbi. (n.d.). Retrieved September 8, 2020, from <https://www.ncbi.nlm.nih.gov/snp/rs1465540247>
33. *Rs147748659 refsnp report—Dbsnp—Ncbi. (n.d.). Retrieved September 8, 2020, from <https://www.ncbi.nlm.nih.gov/snp/rs147748659>
34. *Rs1554331549 refsnp report—Dbsnp—Ncbi. (n.d.). Retrieved September 8, 2020, from <https://www.ncbi.nlm.nih.gov/snp/rs1554331549>
35. *Rs398123301 refsnp report—Dbsnp—Ncbi. (n.d.). Retrieved September 8, 2020, from <https://www.ncbi.nlm.nih.gov/snp/rs398123301>
36. *Rs797045295 refsnp report—Dbsnp—Ncbi. (n.d.). Retrieved September 8, 2020, from <https://www.ncbi.nlm.nih.gov/snp/rs797045295>
37. *Rs750693623 refsnp report—Dbsnp—Ncbi. (n.d.). Retrieved September 8, 2020, from <https://www.ncbi.nlm.nih.gov/snp/rs750693623>
38. *Rs763186740 refsnp report—Dbsnp—Ncbi. (n.d.). Retrieved September 8, 2020, from <https://www.ncbi.nlm.nih.gov/snp/rs763186740> PKHD1
39. Quagga_Rs768427035 refsnp report—Dbsnp—Ncbi. (n.d.). Retrieved September 8, 2020, from <https://www.ncbi.nlm.nih.gov/snp/rs768427035>
40. Rs779852596 refsnp report—Dbsnp—Ncbi. (n.d.). Retrieved September 8, 2020, from <https://www.ncbi.nlm.nih.gov/snp/rs779852596>
41. *Rs-985355103 refsnp report—Dbsnp—Ncbi. (n.d.). Retrieved September 8, 2020, from <https://www.ncbi.nlm.nih.gov/snp/rs985355103>
42. -Schueler, M., Halbritter, J., Phelps, I. G., Braun, D. A., Otto, E. A., Porath, J. D., Gee, H. Y., Shendure, J., O'Roak, B. J., Lawson, J. A., Nabhan, M. M., Soliman, N. A., Doherty, D., & Hildebrandt, F. (2016). Large-scale targeted sequencing comparison highlights extreme genetic heterogeneity in nephronophthisis-related ciliopathies. Journal of medical genetics, 53(3), 208–214. <https://doi.org/10.1136/jmedgenet-2015-103304>
43. -Siebold, L., Dick, A. A., Thompson, R., Maggiore, G., Jacquemin, E., Jaffe, R., Strautnieks, S., Grammatikopoulos, T., Horslen, S., Whitington, P. F., & Shneider, B. L. (2010). Recurrent low gamma-glutamyl transpeptidase cholestasis following liver transplantation for bile salt export pump (BSEP) disease (posttransplant recurrent BSEP disease). Liver transplantation: official publication of the American Association for the Study of Liver Diseases and the International Liver Transplantation Society, 16(7), 856–863. <https://doi.org/10.1002/lt.22074>
44. --Swanson, M. A., Coughlin, C. R., Jr, Scharer, G. H., Szerlong, H. J., Bjoraker, K. J., Spector, E. B., Creadon-Swindell, G., Mahieu, V., Matthijs, G., Hennermann, J. B., Applegarth, D. A., Toone, J. R., Tong, S., Williams, K., & Van Hove, J. L. (2015). Biochemical and molecular predictors for prognosis in nonketotic hyperglycinemia. Annals of neurology, 78(4), 606–618. <https://doi.org/10.1002/ana.24485>
45. --Tuttolomondo, A., Simonetta, I., Duro, G., Pecoraro, R., Miceli, S., Colomba, P., Zizzo, C., Nucera, A., Daidone, M., Di Chiara, T., Scaglione, R., Della Corte, V., Corpora, F., Vogiatzis, D., & Pinto, A. (2017). Inter-familial and intra-familial phenotypic variability in three Sicilian families with Anderson-Fabry disease. Oncotarget, 8(37), 61415–61424. <https://doi.org/10.18632/oncotarget.18250>
46. -Vcv000005314. 4-Clinvar—Ncbi. (n.d.). Retrieved September 9, 2020, from <https://www.ncbi.nlm.nih.gov/clinvar/variation/5314/>
47. --Vcv000166988.8-ClinVar-NCBI (n.d.). Retrieved October 20, 2020, from <https://www.ncbi.nlm.nih.gov/clinvar/variation/166988/>
48. -- Vcv000322941.5-Clinvar—Ncbi. (n.d.). Retrieved September 9, 2020, from <https://www.ncbi.nlm.nih.gov/clinvar/variation/322941/?new_evidence=true>
49. -Waisbren, S. E., Landau, Y., Wilson, J., & Vockley, J. (2013). Neuropsychological outcomes in fatty acid oxidation disorders: 85 cases detected by newborn screening. Developmental disabilities research reviews, 17(3), 260–268. <https://doi.org/10.1002/ddrr.1119>
50. -Wang, N. L., Lu, Y., Gong, J. Y., Xie, X. B., Lin, J., Abuduxikuer, K., Zhang, M. H., & Wang, J. S. (2020). Molecular findings in children with inherited intrahepatic cholestasis. Pediatric research, 87(1), 112–117. <https://doi.org/10.1038/s41390-019-0548-8>
51. --Wendum, D., Barbu, V., Rosmorduc, O., Arrivé, L., Fléjou, J. F., & Poupon, R. (2012). Aspects of liver pathology in adult patients with MDR3/ABCB4 gene mutations. Virchows Archiv : an international journal of pathology, 460(3), 291–298. <https://doi.org/10.1007/s00428-012-1202-6>
52. -Xia, W., Hu, J., Liu, F., Ma, J., Sun, S., Zhang, J., Jin, K., Huang, J., Jiang, N., Wang, X., Li, W., Ma, Z., & Ma, D. (2017). New role of LRP5, associated with nonsyndromic autosomal-recessive hereditary hearing loss. Human mutation, 38(10), 1421–1431. <https://doi.org/10.1002/humu.23285>
53. -Xiong, H. Y., Alipanahi, B., Lee, L. J., Bretschneider, H., Merico, D., Yuen, R. K., Hua, Y., Gueroussov, S., Najafabadi, H. S., Hughes, T. R., Morris, Q., Barash, Y., Krainer, A. R., Jojic, N., Scherer, S. W., Blencowe, B. J., & Frey, B. J. (2015). RNA splicing. The human splicing code reveals new insights into the genetic determinants of disease. Science (New York, N.Y.), 347(6218), 1254806. <https://doi.org/10.1126/science.1254806>
54. -Yang, Q., Yi, S., Li, M., Xie, B., Luo, J., Wang, J., Rong, X., Zhang, Q., Qin, Z., Hang, L., Feng, S., & Fan, X. (2019). Genetic analyses of oculocutaneous albinism types 1 and 2 with four novel mutations. BMC medical genetics, 20(1), 106. <https://doi.org/10.1186/s12881-019-0842-7>
55. -Zhou, Y., & Zhang, J. (2014). Arthrogryposis-renal dysfunction-cholestasis (ARC) syndrome: from molecular genetics to clinical features. Italian journal of pediatrics, 40, 77. <https://doi.org/10.1186/s13052-014-0077-3>
